# Iron Deficiency Anemia as a Rare Risk Factor for Recurrent Pulmonary Embolism and Deep Vein Thrombosis

**DOI:** 10.7759/cureus.13721

**Published:** 2021-03-05

**Authors:** Ebubechukwu Ezeh, Abdulrahman Katabi, Imran Khawaja

**Affiliations:** 1 Internal Medicine, Marshall University Joan C. Edwards School of Medicine, Huntington, USA; 2 Pulmonary Medicine, Marshall University Joan C. Edwards School of Medicine, Huntington, USA

**Keywords:** iron-deficiency, pulmonary embolism (pe), deep venous thrombosis (dvt), primary or autonomous thrombocytosis, systemic anticoagulation, venous thromboembolism (vte)

## Abstract

Iron deficiency is a well-known cause of anemia. However, it is an under-recognized cause of venous thromboembolism (VTE). Iron deficiency predisposes to VTE mainly by inducing thrombocytosis, which can lead to a hypercoagulable state. Identifying iron deficiency as a possible cause of thromboembolic phenomena has clinical significance since this is a potentially avoidable risk factor. This case report serves as a reminder that iron deficiency is an important risk factor for VTE that should be considered in the evaluation of patients. This is particularly true in patients like ours who have recurrent venous thromboembolic disease.

## Introduction

Iron deficiency, the most common nutritional deficiency worldwide, is often associated with reactive thrombocytosis, which can cause a hypercoagulable state [1]. The hypercoagulable state is one of the components of Virchow's triad, which explains the possibility of increasing risk of venous thromboembolism (VTE) associated with it. Many studies have corroborated this association. For instance, within a study group of children with iron deficiency, reactive thrombocytosis was found in up to one-third [2]. Mostly mild, thrombocytosis in iron deficiency anemia (IDA) can become clinically significant by leading to VTEs. This is especially important since detailed workup for VTE usually does not include basic subtle risk factors like IDA. We present a case of recurrent bilateral pulmonary embolism and deep vein thrombosis due to IDA.

This article was previously presented as a meeting abstract at the 2020 Chest Annual Meeting on October 18, 2020.

## Case presentation

A 72-year-old female presented to the emergency room complaining of dizziness and shortness of breath. She was found to be severely anemic and was admitted to the intensive care unit (ICU) with acute blood loss anemia. Past medical history was significant for iron deficiency anemia that was diagnosed five years prior to presentation. Upper endoscopy and colonoscopy at that time were normal. At that time, she was also diagnosed with pulmonary embolism and treated with anticoagulation for about three years which was discontinued due to persistent anemia. On presentation, physical examination was normal. Laboratory data on this admission showed hemoglobin of 4.4 gram/deciliter, mean corpuscular volume (MCV) of 56 femtoliter, red cell distribution width (RDW) of 20%, total iron of 9 microgram/deciliter, iron saturation of 1.9%, total iron binding capacity (TIBC) of 464 microgram/deciliter, and ferritin of 10.6 nanogram/milliliter. Tumor markers including carcinoembryonic antigen (CEA), cancer antigen-125 (CA-125) and CA 19-9 were all within the normal range. Celiac panel was normal. Abdomen and pelvis computed tomography (CT) scan showed bilateral pulmonary embolism (PE) which was confirmed on a chest CT scan. Lower limbs venous duplex showed bilateral deep venous thrombosis (DVT). Upper endoscopy and capsule endoscopy showed normal mucosa and no evidence of bleeding. Testing for Factor V Leiden deficiency, antithrombin and prothrombin abnormalities, protein C and S and mixing studies were inconclusive probably because of blood transfusions and active thromboses. Patient received 4 units of packed RBCs and intravenous iron. An inferior vena cava (IVC) filter was inserted due to concern for bleeding with use of anticoagulation. After the patient’s hemoglobin stabilized and active GI bleeding was ruled out, intravenous heparin was initiated and later transitioned to apixaban upon discharge from the hospital.

## Discussion

Iron deficiency is an underestimated thromboembolic risk factor. Although the secondary thrombocytosis that occurs with IDA is usually considered to be harmless, there is accumulating evidence that elevated platelet counts, especially in the setting of iron deficiency, can lead to an increased thromboembolic risk in both arterial and venous systems [1]. In this regard, several studies have reported various VTE events attributable to IDA. One study reported a case of severe iron deficiency anemia with marked thrombocytosis that was complicated by central retinal vein occlusion [3]. The platelet count was reported to have rapidly declined with improvement in central vein occlusion following the administration of ferrous fumarate. This therapeutic intervention was in support of the fact that IDA contributed to the VTE event [3]. This phenomenon could also explain the association between iron-deficient anemia and reversible focal deficits and stroke found by some authors [4]. A similar outcome was noted in our patient, who had no further episodes of VTE upon treatment of her IDA.

The mechanisms of thrombocytosis and thus VTE in IDA are not completely understood. There are several hypotheses that have tried to explain this association. First, iron is an important regulator of thrombopoiesis [5]. Normal iron levels are required to prevent thrombocytosis by inhibiting thrombopoiesis. Thus, IDA is associated with lack of inhibition of thrombocytosis, which presents a thrombotic risk [6]. Also, because not all cases of iron-related thrombotic events occur in patients with concomitant high platelet count, other pathogenic mechanisms have been proposed. For instance, one such proposed mechanism revolves around the role of iron as an antioxidant. Thus, in addition to the increased thrombotic risk associated with thrombocytosis, some authors have suggested that the decrease in antioxidant defense in iron deficiency anemia may cause increased oxidant stress, which in turn may result in a tendency toward platelet aggregation [7]. Another proposed mechanism is by the altered blood flow pattern evident in iron deficiency. Iron deficiency may contribute to a hypercoagulable state by affecting blood flow patterns within the vessels because of reduced deformability and increased viscosity of microcytic red blood cells [8]. 

Patients with recurrent VTEs undergo extensive workup for VTE that does not usually include basic subtle risk factors like IDA. Thus, we propose that IDA be routinely screened for in patients with unexplained, unprovoked VTEs. This is especially so when there is associated thrombocytosis.

## Conclusions

VTE has been well documented in association with hyperviscosity and polycythemia. There are very few cases reporting thromboembolism due to IDA. A high index of suspicion for VTE should be suspected especially since the symptoms of shortness of breath from PE may overlap with anemia symptoms.

## References

[1] Evstatiev R (2016). Eisenmangel, thrombozytose und thromboembolie (Article in German). Wien Med Wochenschr.

[2] Dickeroff R, von Ruecker A (1991). Thrombozytose im Kindesalter. Differentialdiagnose und klinische Bedeutung (Article in German). Paediatrische Praxis.

[3] Nagai T, Komatsu N, Sakata Y, Miura Y, Ozawa K (2005). Iron deficiency anemia with marked thrombocytosis complicated by central retinal vein occlusion. Intern Med.

[4] Gillum R, Sempos C, Makuc D, Looker AC, Chien CY, Ingram DD (1996). Serum transferrin saturation, stroke incidence, and mortality in women and men. The NHANES I epidemiologic follow-up study. Am J Epidemiol.

[5] Karpatkin S, Garg SK, Freedman ML (1974). Role of iron as a regulator of thrombopoiesis. Am J Med.

[6] Franchini M, Targher G, Montagnana M, Lippi G (2007). Iron and thrombosis. Ann Hematol.

[7] Tekin D, Yavuzer S, Akar N, Cin S (2001). Possible effects of antioxidant status on increased platelet aggregation in childhood iron-deficiency anemia. Pediatr Int.

[8] Hartfield DS, Lowry NJ, Keene DL, Yager JY (1997). Iron deficiency: a cause of stroke in infants and children. Pediatr Neurol.

